# Neutralization of SARS-CoV-2 with IgG from COVID-19-convalescent plasma

**DOI:** 10.1038/s41598-021-84733-5

**Published:** 2021-03-10

**Authors:** Kenji Maeda, Nobuyo Higashi-Kuwata, Noriko Kinoshita, Satoshi Kutsuna, Kiyoto Tsuchiya, Shin-ichiro Hattori, Kouki Matsuda, Yuki Takamatsu, Hiroyuki Gatanaga, Shinichi Oka, Haruhito Sugiyama, Norio Ohmagari, Hiroaki Mitsuya

**Affiliations:** 1 Department of Refractory Viral Infections, National Center for Global Health and Medicine (NCGM) Research Institute, Tokyo, Japan; 2 Disease Control and Prevention Center (DCC), NCGM, Tokyo, Japan; 3 AIDS Clinical Center, NCGM, Tokyo, Japan; 4 Respiratory Medicine, NCGM Center Hospital, Tokyo, Japan; 5 Experimental Retrovirology Section, HIV and AIDS Malignancy Branch, National Cancer Institute, National Institutes of Health, Bethesda, MD USA; 6 Department of Clinical Sciences, Kumamoto University Hospital, Kumamoto, Japan

**Keywords:** SARS-CoV-2, Viral infection, Immunotherapy

## Abstract

While there are various attempts to administer COVID-19-convalescent plasmas to SARS-CoV-2-infected patients, neither appropriate approach nor clinical utility has been established. We examined the presence and temporal changes of the neutralizing activity of IgG fractions from 43 COVID-19-convalescent plasmas using cell-based assays with multiple endpoints. IgG fractions from 27 cases (62.8%) had significant neutralizing activity and moderately to potently inhibited SARS-CoV-2 infection in cell-based assays; however, no detectable neutralizing activity was found in 16 cases (37.2%). Approximately half of the patients (~ 41%), who had significant neutralizing activity, lost the neutralization activity within ~ 1 month. Despite the rapid decline of neutralizing activity in plasmas, good amounts of SARS-CoV-2-S1-binding antibodies were persistently seen. The longer exposure of COVID-19 patients to greater amounts of SARS-CoV-2 elicits potent immune response to SARS-CoV-2, producing greater neutralization activity and SARS-CoV-2-S1-binding antibody amounts. The dilution of highly-neutralizing plasmas with poorly-neutralizing plasmas relatively readily reduced neutralizing activity. The presence of good amounts of SARS-CoV-2-S1-binding antibodies does not serve as a surrogate ensuring the presence of good neutralizing activity. In selecting good COVID-19-convalescent plasmas, quantification of neutralizing activity in each plasma sample before collection and use is required.

## Introduction

SARS-CoV-2 causes severe, acute, and often fatal diseases in humans and is considered a global public threat^1–4^. As of November 7, 2020, more than 48.5 million COVID-19 cases have been reported in over 200 countries and more than 1,230,000 people have died^3^. Moreover, SARS-CoV-2 may persist in some convalescent COVID-19 survivors for long periods of time so that it is possible that SARS-CoV-2 infection could continue to recur, leading to the persistence of the pandemic^5^. Thus, development of effective vaccine(s) and specific therapeutics active against SARS-CoV-2 is of high priority; however, it may take at least a year or more to develop such effective vaccines and specific therapeutics. Since passive transfer of antibodies has been shown to protect non-human primates from the lethal challenges with Ebola virus, such plasma therapy has been used to treat patients with Ebola virus disease, severe acute respiratory syndrome (SARS) and H1N1 influenza^6–9^. Some anecdotal studies suggest some successes in mitigating symptoms and decreasing the number of deaths from such diseases including COVID-19^8,10–12^, but its approaches and potential efficacy have not been examined in rigorous clinical trials. Moreover, the possible adverse effects with such plasma transfer regarding SARS-CoV-2 infection have not been elucidated. Nevertheless, on August 23, 2020, the Federal Food and Drug Administration of the United States issued an Emergency Use Authorization (EUA) for emergency use of COVID-19 convalescent plasma for the treatment of hospitalized patients with COVID-19^13^.

Thus, in the present study, in order to examine the presence and persistence of neutralizing activity in the plasma or serum samples obtained from 43 COVID-19-convalescent cases employing cell-based assays and various virologic endpoints. We found that certain IgG fractions from convalescent plasma or serum samples completely inhibited the infectivity, replication, and cytopathicity of SARS-CoV-2; however, the strength of neutralization significantly varied from one case to another. Of the 43 COVID-19-convalescent patients, 16 (37.2%) had no detectable neutralizing activity throughout the clinical course. Sixteen (40.7%) of the 27 patients who had neutralizing antibodies with neutralizing activity lost the activity within ~ one month (range 2–7 weeks), which may be related to viral reactivation, re-infection, or else^5,14^. The present data strongly suggest that neutralizing activity of plasma should be titrated prior to plasma collection and neutralizing plasma should be collected soon after the determination of neutralizing activity and that only plasma that contains good amounts of neutralizing antibodies should be administered to patients with COVID-19.

## Results

### Clinical characteristics

Forty-three patients, RNA-PCR-confirmed, diagnosed to have COVID-19, and admitted to the Center Hospital of the National Center for Global Health & Medicine, participated in the present study. The median age of the patients was 53 (ranging from 28 to 83 years old). Three of the 43 patients showed minor symptoms at the onset (shown to be RNA-PCR test positive) and recovered shortly. Of the 43 patients, 21 (48.8%) had hypoxia and were diagnosed with pneumonia. Seven patients with pneumonia required positive-pressure ventilation, two of whom were also treated with ECMO (extracorporeal membrane oxygenation). All patients except for the two ECMO-treated cases recovered after 2 to 6 weeks of oxygen administration. Clinical profiles of patients enrolled in this study are shown in Table 1 and Supplementary Table S1.

**Table 1:** Summary of the patient characteristics.

Disease severity^*1^*^1^	All patients (n = 43)	Mild (n = 3)	Moderate (n = 19)	Severe (n = 14)	Critical (n = 7)
**Characteristics**					
Age, years	53 (28–83)	44 (29–70)	47 (28–71)	53 (33–83)	63 (40–78)
**Sex**					
Men	35 (81.4)	3 (100.0)	15 (78.9)	10 (71.4)	7 (100.0)
Women	8 (18.6)	0 (0.0)	4 (21.1)	4 (28.6)	0 (0.0)
**Clinical course**					
*Respiratory support*					
None	22 (51.2)	3 (100.0)	19 (100.0)	0 (0.0)	0 (0.0)
Oxygen inhalation	21 (48.8)	0 (0.0)	0 (0.0)	14 (100.0)	7 (100.0)
Mechanical ventilation	7 (16.3)	0 (0.0)	0 (0.0)	0 (0.0)	7 (100.0)
ECMO*^2^	2 (4.7)	0 (0.0)	0 (0.0)	0 (0.0)	2 (28.6)
**Pneumonia**					
Yes	40 (93.0)	0 (0.0)	19 (100.0)	14 (100.0)	7 (100.0)
No	3 (7.0)	3 (100.0)	0 (0.0)	0 (0.0)	0 (0.0)
**Outcome**					
Discharged	40 (93.0)	3 (100.0)	19 (100.0)	14 (100.0)	4 (57.1)
Deceased	3 (7.0)	0 (0.0)	0 (0.0)	0 (0.0)	3 (42.9)
Neutralizing activity*^3^					
Yes	27 (62.8)	0 (0.0)	9 (47.4)	12 (85.7)	6 (85.7)
No	16 (37.2)	3 (100.0)	10 (52.6)	2 (14.3)	1 (14.3)

*^1^Disease severity definitions. Mild: febrile or fatigue but no pneumonia identified; Moderate: febrile, fatigue, moderate pneumonia identified, but no dyspnea and no oxygen inhalation required; Severe: febrile, fatigue, dyspnea, severe pneumonia identified, and oxygen inhalation required; Critical: febrile, fatigue, severe dyspnea, critical pneumonia identified, and positive pressure ventilation plus extracorporeal membrane oxygenation (ECMO*^2^) required. *^3^For the detail of neutralizing activity, see Fig. 3A.

### Neutralizing activity of IgG fractions from COVID-19-convalescent plasma

**Figure 1:**
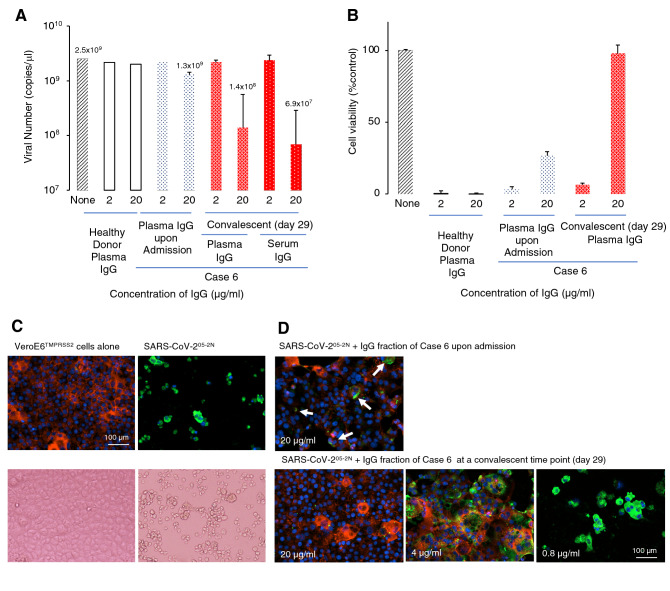
Neutralizing activity of IgG fraction from a representative convalescent patient. (**A**) SARS-CoV-2^05-2N^ was mixed with the IgG fraction from a patient (Case 6) or a healthy donor, incubated for 20 min, and then inoculated to VeroE6^TMPRSS2^ cells. The infectivity of the virus was examined by determining viral copy numbers using RNA-PCR. (**B**) The inhibition of cytopathic effect of SARS-CoV-2^05-2N^ in VeroE6^TMPRSS2^ cells by the IgG fraction from the plasma of Case 6 or a healthy donor. (**C**,**D**) Immunocytochemistry of SARS-CoV-2^05-2N^-infected VeroE6^TMPRSS2^ cells. The IgG fraction (20 µg/ml) from Case 6 completely blocked the infectivity and cytopathic effect of SARS-CoV-2 and no cells got stained in green. Upper and lower insets in Panel (**D**) represent merged images, which are composed of images obtained from 3-color fluorescence. Viral antigens, actin filaments, and nuclei are indicated in green, red, and blue. The upper inset in (**D**) shows that most VeroE6^TMPRSS2^ cells were protected by the IgG fraction (20 µg/ml), while a few cells were infected (indicated by arrows). Lower right panel shows that the structure of actin filaments was totally destroyed by SARS-CoV-2^05-2N^ infection and almost all the cells got stained in green (SARS-CoV-2-positive); however, 20 µg/ml of the IgG fraction from Case 6 completely blocked the infectivity and cytopathicity of the virus. Images of immunocytochemistry were obtained using Cytation5 (BioTek, VT).

IgG fractions were obtained from plasma or serum from 43 COVID-19 patients and their neutralizing activity was determined using TMPRSS2-overexpressing VeroE6 (VeroE6^TMPRSS2^) cells^15^ and SARS-CoV-2^05-2N^ that was isolated from Case 1. Plasma or serum samples were collected from each patient upon admission and on various timepoints (upon discharge or following the recovery or discharge). In some patients, additional samples were obtained for detailed time-course analyses. In neutralization assays, a portion of SARS-CoV-2^05-2N^ at an MOI of 0.01 was mixed with various amounts of purified IgG fraction from each of COVID-19 patients or healthy donors and the mixture was inoculated to VeroE6^TMPRSS2^ cells that had been overnight seeded onto microtiter culture plates and cultured for 3 days. The neutralizing activity of each IgG fraction was examined using the reduction in the number of viral copies in the supernatants (determined with RNA-PCR method)^16^, the reduction of cytopathic effects (CPE), and immunocytochemistry. Figure 1A–D show the neutralizing activity of IgG fraction from Case 6. The IgG fraction from Case 6′s plasma upon admission (day 10 from the onset of disease), at a concentration of 20 µg/ml, exerted slight to moderate suppression on viral production (Fig. 1A) and CPE in VeroE6^TMPRSS2^ cells (Fig. 1B). By contrast, the IgG fraction obtained from plasma and serum of Case 6 at the time of discharge (day 29 from the onset) reduced viral copy numbers by more than 1 log_10_ at 20 µg/ml (Fig. 1A, the very right column) and virtually completely blocked the cytopathicity of SARS-CoV-2^05-2N^ at 20 µg/ml (Fig. 1B, the very right column). The neutralizing activity of IgG fraction from Case 6 was also examined using immunocytochemistry. The upper left inset in Fig. 1C shows a representative immunocytochemistry image of VeroE6^TMPRSS2^ cells cultured alone, depicting robust actin filaments in red and nuclei of healthy cells in blue. The lower left inset in Fig. 1C shows an image under inverted light microscopy, where robust live VeroE6^TMPRSS2^ cells are seen. However, the VeroE6^TMPRSS2^ cells exposed to SARS-CoV-2^05-2N^ and cultured alone were substantially destroyed and the actin filaments were almost totally lost due to rearrangements caused by the virus^17^ and most of the cells had detached from the microtiter culture plates (upper and lower right insets in Fig. 1C). The IgG fraction upon admission, at 20 µg/ml, substantially protected VeroE6^TMPRSS2^ cells but permitted viral breakthrough (indicated by arrows in upper inset in Fig. 1D). However, the IgG fraction obtained upon discharge of Case 6, at 20 µg/ml, completely blocked the infectivity and cytopathicity of SARS-CoV-2^05-2N^ (lower very left inset in Fig. 1D), corroborating the results shown in Fig. 1A,B. Virtually the same features were seen as examined with immunocytochemistry using another cell line (VeroE6 cells) (Supplementary Fig. S1).

### SARS-CoV-2-neutralizing activity and SARS-CoV-2-S1-binding antibody amounts in IgG fractions from COVID-19-convalescent patients

**Figure 2:**
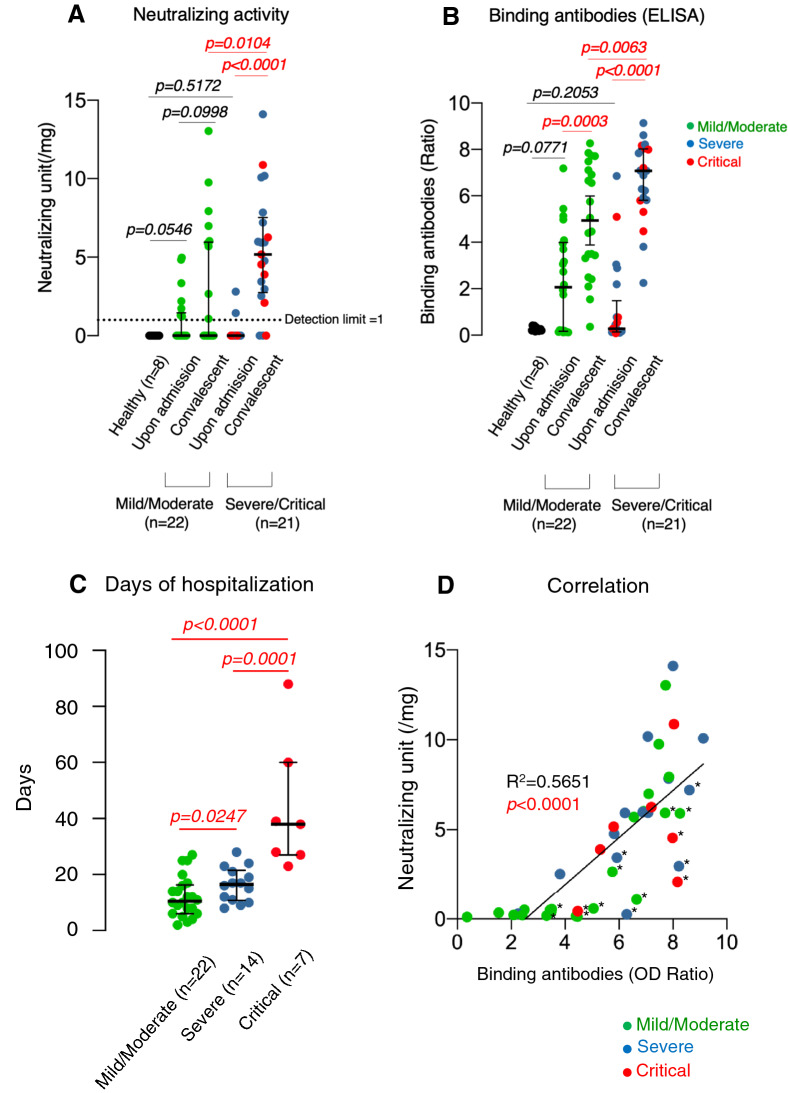
Changes in neutralizing activity of IgG fractions and the amounts of SARS-CoV-2-S1-binding antibodies. (**A**,**B**) IgG fractions from plasma (or serum) samples of all the 43 patients obtained upon admission and during convalescence were tested for their neutralizing activity and SARS-CoV-2-S1-binding antibody levels. The neutralizing activity of each IgG fraction was normalized by the activity of 100 µg IgG fraction of a patient D008 obtained at a convalescence time point, referred as to be 1 neutralization unit (Supplementary Fig. S2). The dashed line (1 neutralizing unit/mg) denotes the detection limit. The amounts of SARS-CoV-2-S1-binding IgG were measured using ELISA and shown by the ratios between the extinction of the sample and the calibrator (See “Materials and Methods” section for more details on the calculation. For disease severity definitions (Mild/Moderate: n = 22, Severe/Critical: n = 21), see the footnote in Table 1. *p*-value: Mann–Whitney U test. (**C**) Correlation of disease severity (Mild/Moderate: 22, Severe: 14, Critical: 7) and days of hospitalization. (**D**) Correlation of the SARS-CoV-2 binding IgG amounts and the neutralizing activity. Statistical analysis was performed and figures were generated using GraphPad Prism software version 8 (https://www.graphpad.com/scientific-software/prism/) (La Jolla, CA).

We then determined SARS-CoV-2-neutralizing activity and SARS-CoV-2-S1-binding antibody amounts^18^ in IgG fractions from all the 43 patients upon admission and during convalescent periods. Based on the severity of clinical profiles of COVID-19, the patients were categorized into 4 groups: mild, moderate, severe, and critical groups (See categorization definitions in the footnote to Table 1 and Supplementary Table S1). Figure 2A,B show neutralizing activity and SARS-CoV-2-S1-binding antibody amounts upon admission, respectively. Figure 2A,B also show the highest neutralizing activity levels and the highest SARS-CoV-2-S1-binding antibody amounts documented in those with mild/moderate and severe/critical symptoms during convalescent periods. A few patients had greater neutralizing activity upon admission than in healthy volunteers (Fig. 2A), but the difference was not statistically significant (*p* = 0.0546). Nine of the 22 patients with mild or moderate symptoms in their clinical courses, had relatively high neutralizing activity and 13 of the 22 patients had lower-than-detection-limit neutralizing activity, and the 22 patients did not have significantly greater neutralizing activity than upon admission (*p* = 0.0998). In contrast, those with severe/critical symptoms (n = 21) had significantly greater neutralization activity than upon admission (*p* < 0.0001). Of note, patients with severe/critical symptoms had significantly greater neutralization activity than those with mild/moderate symptoms (*p* = 0.0104). Patients with mild/moderate (n = 22) and those with severe/critical symptoms (n = 21) on admission did not have significantly greater amounts of SARS-CoV-2-S1-binding antibody as compared to healthy individuals (n = 8) (*p* = 0.0771 and 0.2053, respectively); however, both groups had significantly greater amounts of SARS-CoV-2-S1-binding antibody during convalescent periods than upon admission (*p* = 0.0003 and *p* < 0.0001, respectively). Notably, patients with severe/critical symptoms had significantly greater SARS-CoV-2-S1-binding antibody amounts than patients with mild-moderate symptoms (*p* = 0.0063).

We then examined the correlation of the clinical severity of infected patients with their hospitalization lengths. As shown in Fig. 2C, patients with severe symptoms had significantly longer hospitalization than those with mild/moderate symptoms (*p* = 0.0247). Moreover, patients with critical symptoms had further longer hospitalization than those with severe symptoms (*p* = 0.0001). We also examined the correlation between neutralization activity and SARS-CoV-2-S1-binding antibody amounts in all the 43 COVID-19 patients. Figure 2D demonstrates a significant correlation between the two variables (*p* < 0.0001); however, there were a substantial number of individuals with considerably lower neutralization activity relative to their SARS-CoV-2-S1-binding antibody amounts, who are plotted under the correlation line and indexed by asterisks in Fig. 2D.

### Neutralizing activity diminishes in as soon as 1–2 months in various COVID-19 patients

**Figure 3:**
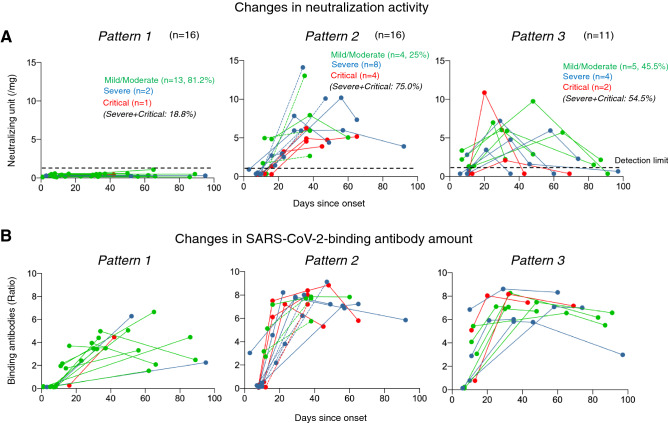
Changes in neutralizing activity and SARS-CoV-2-S1-binding antibody levels. (**A**) Neutralizing activity of each IgG fraction and the amount of SARS-CoV-2-binding antibody in serum/plasma obtained from multiple time points in the 43 patients were determined. The neutralizing activity of each IgG fraction was normalized and presented as a neutralizing unit, as described in the legend to Fig. 2. The definition of Patterns [Pattern 1, in which no neutralizing activity was observed during observation periods; Pattern 2, in which the neutralizing activity was maintained during observation periods, or cases with two time-point data only (shown with dashed line); Pattern 3, in which neutralizing activity was lost after the peak of the activity by < 40% during observation periods]. For the neutralizing activity in Pattern 3 (upper right panel), the average time period from the disease onset to reach the peak of activity was 36 days, and the average time period it took for the same individual to lose the activity by 50% or more from the peak was 24 days. The dashed line in the three upper panels denotes the detection limit value (1 neutralization unit). (**B**) Changes in SARS-CoV-2-S1-binding antibody amounts in patients in each Pattern group. For the definition of severities of patients, see the footnote to Table 1.

When we determined neutralization activity in all the 43 COVID-19 patients at multiple time points; upon admission, during hospitalization, and after discharge, we identified three distinct patterns in temporal changes in neutralization activity: (i) Pattern 1, in which no significant neutralizing activity was seen (all under-detection-limit) throughout our observation periods, (ii) Pattern 2, in which substantial neutralization activity was seen by day 20–30 after disease onset and/or persisted (did not diminish beyond 50%) during our observation periods up to 92 days after disease onset, and (iii) Pattern 3, in which significant neutralization activity was seen but decreased by > 60% within 9–50 days (average 24 days) after the peaks seen in neutralization. Sixteen (37.2%), 16 (37.2%), and 11 (25.6%) of the 43 patients were in Patterns 1, 2, and 3, respectively. Interestingly, the majority of the patients in Pattern 1 (13/16; 81.2%) was with mild/moderate symptoms, while the majority of patients in Pattern 2 (12/16; 75%) was with severe/critical symptoms. In contrast, a half of the patients who were in Pattern 3 were with mild/moderate (45.5%) and other half of the patients with severe/critical symptoms (54.5%). When we examined the changes in SARS-CoV-2-S1-binding antibody amounts in the patients in Pattern 1, all of those had substantial amounts of SARS-CoV-2-S1-binding antibodies but had no significant neutralization activity (all under the detection-limit). Patients who belonged to Pattern 2 generally had further greater amounts of the SARS-CoV-2-binding antibodies and the high amounts of such antibodies persisted throughout the observation periods or only gradually declined by 34%. All the patients in Pattern 3 generally developed moderate to high neutralization activity during their clinical courses and such high neutralization activity decreased by greater than 50% or down to undetectable levels; however, all of those in Pattern 3 continuously had SARS-CoV-2-binding IgG antibodies (right panel in Fig. 3B). These data suggest that in SARS-CoV-2-infected individuals, SARS-CoV-2-neutralizing activity diminishes relatively sooner compared to total SARS-CoV-2-binding IgG antibodies.

### Neutralizing activity of convalescent plasma IgG fractions varies depending on individuals and does not always correlate with the amounts of SARS-CoV-2-S1-binding antibodies

**Figure 4:**
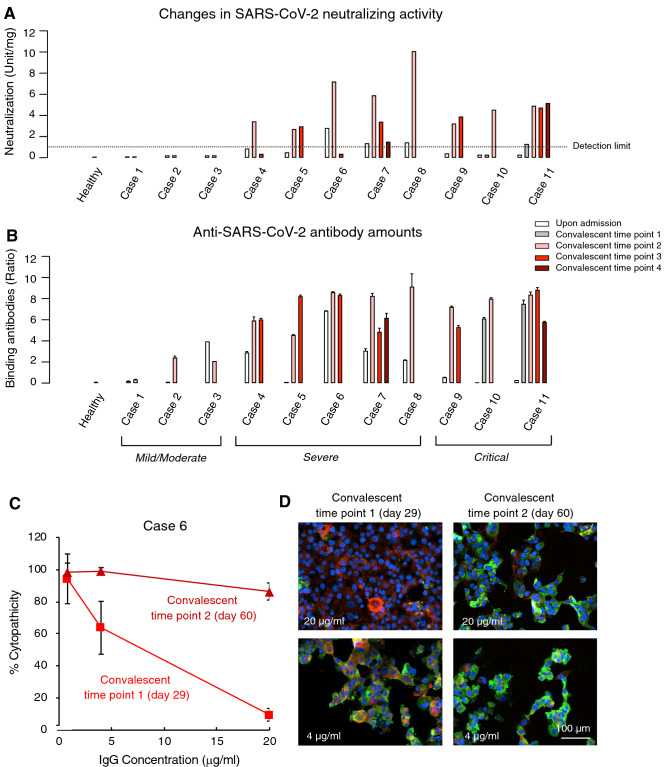
Comparison of neutralizing activity of IgG fractions with SARS-CoV-2-S1-binding IgG levels and changes in neutralizing activity. (**A**) The neutralizing activity of each IgG fraction was normalized and shown as a neutralizing unit. The broken line denotes the detection limit (1 neutralization unit). Regarding convalescence time points, 1 to 4, see “days of sample collections” in Table S1. (**B**) The amounts of SARS-CoV-2-S1-binding IgG were measured using ELISA and shown by the ratios between the extinction of the sample and the calibrator. See Materials and Method section for more details. (**C**) Loss of neutralizing activity of IgG fraction from Case 6 at Convalescence time point 2 (60 days from the onset). Note that the potent neutralization activity of IgG fraction from Case 6 observed at Convalescent time point 1 (29 days from the onset) was lost by Convalescent time point 2. (**D**) Immunocytochemistry of VeroE6^TMPRSS2^ cells exposed to SARS-CoV-2^05-2N^ and cultured in the presence of IgG fraction from Case 6. Note that while the cells were virtually completely protected by IgG fraction (20 µg/ml) at Convalescence time point 1, such activity was significantly reduced and virtually all the cells were infected and stained in green. Cellular actin filaments, SARS-CoV-2^05-2N^ antigens, and cell nuclei stained in red, green, and blue, respectively. Graphs were generated using GraphPad Prism software version 8.

As shown above, neutralizing activity of convalescent plasma IgG fractions varies depending on individuals and does not always correlate with the amounts of SARS-CoV-2-S1-binding antibodies (Fig. 2D) and that neutralizing activity diminishes sooner than SARS-CoV-2-S1-binding antibodies in a substantial number of the infected individuals (Fig. 3A). Thus, we examined neutralization activity and total SARS-CoV-2-S1-binding IgG antibody amounts on multiple time points in more details in 11 selected COVID-19 patients. Three selected patients with mild/moderate symptoms (Cases 1–3 in Fig. 4) had no detectable neutralization activity in their IgG fractions as examined using two endpoints: (i) % inhibition of the cytopathicity of SARS-CoV-2^05-2N^ in VeroE6^TMPRSS2^ cells by IgG fraction from each patient (Fig. 4A and Supplementary Fig. S3A) and (ii) changes in viral copy numbers in the culture supernatants of VeroE6^TMPRSS2^ cells exposed to SARS-CoV-2^05-2N^ in the presence of various concentrations of IgG (Supplementary Fig. S3B). When SARS-CoV-2-binding antibody amounts in those 3 patients (Cases 1–3) were determined using the ELISA method, low to relatively moderate amounts of antibodies were identified (Fig. 4B).

Five selected patients with severe symptoms (Cases 4–8 in Fig. 4) had under-detection-limit or marginal neutralization activity upon admission and moderate to high-level neutralization activity at convalescent time point 2 (shown in pink; Fig. 4A, and Supplementary Figs. S3 and S4). Of note, Case 5′s moderate neutralization activity stayed the same by convalescent time point 3 (shown in red); however, the activity in Case 6 became under detection limit by convalescent time point 3 (Fig. 4A). The activity in Case 7 was moderate at convalescent time point 2 (in pink), while it decreased by 57% by convalescent time point 3 (in red), and it further reduced close to the detection limit by convalescent time point 4 (shown in brown; Fig. 4A). In contrast, significant amounts of SARS-CoV-2-S1-binding IgG antibodies were seen in all Cases 4–8 by convalescent time point 2 and those amounts generally persisted throughout the observation periods (Fig. 4B). Another three patients with critical symptoms (Cases 9–11 in Fig. 4) had moderate neutralization activity when examined at convalescent time point 2, which persisted throughout all convalescent time points (Fig. 4A). These three patients also had substantial SARS-CoV-2-binding antibody amounts as examined at convalescent timepoints 1 or 2 and those amounts persisted throughout the observation periods (Fig. 4B).

These data reveal that the majority of the patients with mild/moderate symptoms tend not to develop significant neutralizing activity although they develop low to moderate amounts of SARS-CoV-2-binding IgG antibodies throughout convalescent periods and that the majority of those with severe and critical symptoms develop moderate to high-level neutralizing activity and high-level SARS-CoV-2-binding antibodies. The data also demonstrate that some of those with severe/critical symptoms (40.7%: 11 of 27 who had significant neutralizing activity during clinical courses) lose their neutralizing activity during convalescent periods or after discharge.

### No evidence of antibody-dependent-enhanced SARS-CoV-2 infection in vitro

Finally, we examined whether COVID-19-convalescent IgG fractions elicited the antibody-dependent enhanced SARS-CoV-2 infection^19,20^. We chose an IgG fraction from Case 11, in whom his IgG fraction upon admission had no neutralizing activity or SARS-CoV-2-S1-binding antibodies as well as an IgG fraction from Case 8, in whom low to moderate neutralizing activity was identified upon admission (Supplementary Figs. S3 and S4). VeroE6^TMPRSS2^ cells were exposed to the mixture of SARS-CoV-2^05-2N^ at MOIs of 0.0025 and 0.01 and each IgG fraction upon admission at concentrations of 0.8, 4, and 20 µg/ml, were cultured for 3 days, and the viral copy numbers in the supernatants were determined. As shown in Supplementary Fig. S5A, no significant enhancement of SARS-CoV-2^05-2N^ infection was seen when VeroE6^TMPRSS2^ cells were cultured in the presence of IgG fraction of Case 11 at concentrations tested (0.8, 4 and 20 µg/ml) and the numbers of viral RNA were determined. When Case 8′s IgG was examined at low concentrations 0.8 and 4 µg/ml, there was no significant enhancement of infection observed or at the highest concentration, 20 µg/ml. Case 8′s IgG fraction significantly suppressed the infection and reduced the viral copy numbers by ~ 1 log. The presence or absence of enhanced infection was further examined by immunostaining the VeroE6^TMPRSS2^ cells cultured in the presence of a wide range of IgG concentrations Case 11′s IgG fraction failed to block the infection at 0.02 and 0.2 µg/ml, while it partially suppressed the cells from the infection at 2 µg/ml and completely blocked the infection and cytopathicity of SARS-CoV-2^05-2N^ (Supplementary Fig. S5B), showing no evidence for the antibody-dependent enhanced SARS-CoV-2 infection in the present study.

## Discussion

Various attempts have been made to transfer convalescent plasma to treat patients with SARS or MERS; however, neither appropriate approaches nor clinical utilities have been established^7,8^. In the absence of effective and safe vaccines or efficacious therapeutics and with a forecast of persistence of SARS-CoV-2 over substantially longer periods of time, COVID-19-convalescent plasma transfer therapy has been pursued in various nations and a number of institutions. Indeed, multiple clinical trials are already being conducted^8,12^ and certain anecdotal studies suggest some successes in mitigating COVID-19 symptoms and decreasing the number of deaths from COVID-19^8,10–12^, but its approaches and efficacy have not been yet examined in rigorous clinical trials^7,8^. According to a protocol issued by the US Food and Drugs Administration, the plasmas are drawn from convalescent COVID-19 patients, who tested negative for SARS-CoV-2 or who have shown no clinical symptoms for 28 days, and such donors are eligible to provide plasma every 28 days^13^. The collected plasma will be mixed, frozen, and pooled, and are to be used for the treatment of multiple patients. However, the evidence of the efficacy of such therapy is still absent and plasma transfusion therapy might pose serious risks such as transfusion-related acute lung injury (TRALI), allergic reactions, and antibody-dependent enhancement (ADE) of infection by the very virus^20^.

In the most recently publicized data from an open-label phase II multi-center randomized controlled trial, convalescent plasma, whose neutralizing activity had not been determined before transfusion, was administered in two doses (200 ml each) to 235 adult patients with moderate COVID-19, while 229 control patients had only best standard care. That clinical trial demonstrated no reduction in progression to severe COVID-19 or mortality^21^. A similar open-label, multi-center randomized clinical trial, examining 103 patients with severe or life-threatening COVID-19, conducted in Wuhan, China, also did not reveal significant improvement^22^. In contrast, a retrospective study involving 138 patients, who received convalescent plasma provided evidence of clinical improvement^23^. However, in any of these studies, neutralizing antibody titers in the plasma administered had not been determined before transfusion, while transfusion of COVID-19-convalescent plasma, in general, appears to be safe in hospitalized patients with COVID-19^24^. Such an absence of neutralizing antibody titers in the convalescent plasma are highly likely associated with the conflicting study results from clinical trials using COVID-19-convalescent plasma.

In the present study, we examined the presence and persistence of the neutralizing activity of IgG fractions from 43 COVID-19-convalescent cases, quantitative and temporal correlations among the neutralizing activity and SARS-CoV-2-S1-binding antibody amounts in plasma samples and patients’ clinical severities and hospitalization lengths. Our examinations strongly suggested that patients experiencing severe/critical symptoms and longer hospitalization had significantly greater neutralizing activity and SARS-CoV-2-S1-binding antibody amounts than those having mild/moderate symptoms and shorter hospitalization periods, suggesting that the longer exposure of COVID-19 patients to greater amounts of SARS-CoV-2 elicits greater immune response to the virus, producing greater neutralization activity and SARS-CoV-2-S1-binding antibody amounts. However, there should be multiple factors which are associated with the length of incubation periods following the initial exposure to the virus, clinical severities and outcomes, including systemic immune response of the patient to SARS-CoV-2^25^, risk factors such as immunocompromising illnesses, obesity, and pulmonary diseases^26^. Indeed, among those categorized in Pattern 1, there was a patient, who had undergone renal transplantation and was receiving methylprednisolone and immunosuppressants. This patient had critical symptoms (i.e., severe pneumonia) and received invasive positive-pressure ventilation following intratracheal intubation; however, eventually died of ventilator-associated pneumonia. This particular patient, despite of critical symptoms and long hospitalization, never had a detectable level of neutralizing activity in the IgG fraction of his plasma throughout the clinical course (indicated by a red asterisk in Fig. 3A left panel).

Intriguingly, all the 11 patients who fell in Pattern 3 lost their neutralization activity by up to 60% and six of those 11 patients had neutralization reduction down to under the detection limit within 9–50 days (average 24 days) after the peaks in neutralization activity were reached (Fig. 3A, right panel), although there were notable variabilities in the rates and paces in the reduction of neutralization activity in those with Patterns 2 and 3 (Fig. 3). Rapid declines of neutralizing activity after primary viral infection have also been seen in other viral infections. For example, following primary respiratory syncytial virus (RSV) infection in a birth cohort, a rapid decline of neutralizing activity to pre-infection levels has been seen as early as 1–1.9 months post-infection^27^. In the cases of Dengue virus (DENV) infection, some children with primary DENV reportedly had substantial reduction in neutralization activity between convalescent periods and 6 months^28^. The mechanisms of the rapid reduction in neutralizing activity in these viral infections are not presently well understood. In any event, the current results we have obtained demonstrate that the primary neutralizing antibody response against SARS-CoV-2 tends to be short-lived and may at least partially contribute to the susceptibility to re-infection by SARS-CoV-2 as can be seen in the cases of RSV and DENV infections. One of the most important findings in the present study is that despite the rapid decline of neutralizing activity in plasma samples in patients under Pattern 3, those patients persistently had good amounts of SARS-CoV-2-S1-binding antibodies. This finding strongly suggests that the presence of good amounts of SARS-CoV-2-S1-binding antibodies does not serve as a surrogate indicating the presence of good neutralizing activity in patients or in their plasmas. At present, no monoclonal SARS-CoV-2-S1-binding antibody or antibodies that ensure the presence of a full spectrum of SARS-CoV-2-neutralizing antibodies are not available. Thus, in selecting COVID-19-convalescent plasma that are expected to be effective, determining and quantifying neutralizing activity in each plasma sample is required.

Of note, although the IgG fraction from Case 6 showed potent anti-SARS-CoV-2 activity and completely blocked the infectivity and cytopathicity of the virus at 20 µg/ml (Fig. 1D), just fivefold reduction of the IgG concentration to 4 µg/ml substantially reduced its activity, and further fivefold reduction to 0.8 µg/ml completely eliminated the neutralization activity. This finding strongly suggests that pooling highly-neutralizing plasmas and poorly-neutralizing plasmas can easily result in the dilution of neutralizing activity and loss of efficacy.

The current data also imply that the efficacy of anti-SARS-CoV-2 vaccines such as the kinetics and multitude of neutralizing antibody elicited might substantially differ from one individual to another and that the effects of the vaccines might be short-lived and such vaccines might have to be given multiple times. Most importantly, our results indicate the importance of the determination of neutralizing activity of each plasma sample before collection and administration. However, there are several limitations to our present analysis. The methods employed in the present study did not consider cell-mediated immunity per se or the capacity of anti-SARS-CoV-2 antibody to enhance cell-mediated immune response. Moreover, most of the subjects participated in the present study sought medical attention only when they had symptoms due to SARS-CoV-2 infection. Thereby, there are differences between the dates of speculated disease onset and the dates of admission. Therefore, there might be differences in antibody kinetics, which may be associated with clinical sequels.

In the present study, we examined the neutralizing activity of IgG fractions isolated from sera of COVID-19-convalescent patients. It is known that serum levels of IgM and IgA that bind to SARS-CoV-2 virions also increase after viral infection^29^. It is likely that such SARS-CoV-2-binding IgM and IgA are involved in certain protection of the host against the virus; however, the magnitude of antiviral activity of such IgM and IgA relative to that of SARS-CoV-2-binding IgG remains to be elucidated.

To the best of our knowledge, no antibodies that bind to components of infectious SARS-CoV-2 other than its spike glycoprotein and exert neutralizing activity are known to exist, although a minority of human mAbs that bind to the N-terminal domain (NTD) of the S protein have been found^30^. These antibodies inhibit the infectivity of certain SARS-CoV-2 isolates in part through inhibiting a post-attachment step in the infection cycle and require Fc effector functions for optimal neutralization^30^. Thus, a subset of NTD-specific antibodies may leverage neutralizing and Fc-mediated activities to protect against SARS-CoV-2.

As for the antigenic epitopes on the spike glycoprotein of SARS-CoV-2, which are associated with neutralization by anti-SARS-CoV-2 antibodies, Zost et al*.* have identified multiple human monoclonal antibodies (mAbs) that fully blocked the receptor-binding domain (RBD) of the spike glycoprotein from interacting with human ACE2 and exerted potent neutralizing activity against the virus^31^. Competition-binding, structural, and functional studies have allowed clustering of the mAbs into classes recognizing distinct epitopes on the RBD and distinct conformational states of the spike trimer. However, at present, the exact number of antigenic epitopes associated with neutralization is not known. Moreover, it remains to be elucidated as to which epitopes are involved in greater or lesser magnitudes of neutralization so that effective humoral immunity eliciting vaccine and robust immunotherapeutics are well designed^32^.

In order to examine whether anti-SARS-CoV-2 antibody-dependent enhancement (ADE) of SARS-CoV-2 infection occurs, we employed VeroE6 cells and identified no detectable ADE effects (Supplementary Fig. S5). However, the VeroE6 cells we employed in the current study are negative for Fcγ receptor (FcγR: data not shown) and may not serve as appropriate cells for the examination of ADE. FcγR-mediated entry of infectious dengue virus immune complexes into monocytes/macro-phages is hypothesized to be a key event in the pathogenesis of complicated dengue fever, while the mechanism of DENV immune complex internalization may occur using different FcγRs and remains to be determined^33^. In this regard, in contrast to DENV infections, FcγR-expressing cells such as macrophages reportedly do not sustain SARS-CoV infection^34^. While some studies have discussed a correlation between high anti-SARS-CoV IgG titers with the severity of the disease, such studies have failed to show a causal link between the high anti-SARS-CoV IgG titers and the severity of the disease^35^. Moreover, the most recent study of the safety of 5000 patients receiving COVID-19-convalescent plasma suggest that the use of convalescent COVID-19 may not pose clinical deterioration and/or worse outcomes and the ADE may be clinically inconsequential^36^; however, we need to proceed in strict vigilance in the therapy with COVID-19-convalescent plasma. Moreover, more detailed characterization of the immune response and clinical outcomes with the use of neutralizing antibodies and cell-mediated immunity should be required in furthering our understanding of the antibody response after SARS-CoV-2 infection, which should aid the design of effective COVID-19-convalescent plasma therapy and provide insights in the strategy to develop anti-COVID-19 vaccines.

## Methods

### Patients

Forty-three patients, who were clinically diagnosed with COVID-19 and were admitted to the National Center for Global Health and Medicine (NCGM) in Tokyo, were enrolled in this study. All patients were confirmed to be SARS-CoV-2-positive using RNA-PCR with nasopharyngeal swabs at the time of study enrollment. The Ethics Committee at the NCGM approved the present study (NCGM-G-003472-02), each patient provided a written informed consent, and this study abided by the Declaration of Helsinki principles.

### Cells, viruses, and isolation of IgG fractions from COVID-19-convalescent patients

VeroE6^TMPRSS2^ cells^15^ and its parental VeroE6 cells were obtained from Japanese Collection of Research Bioresources (JCRB) Cell Bank (Osaka, Japan). VeroE6^TMPRSS2^ cells were maintained in D-MEM supplemented with 10% FCS, 100 µg/ml of penicillin, 100 µg/ml of streptomycin, and 1 mg/mL of G418. VeroE6 cells were maintained in the D-MEM culture medium above mentioned but without G418. SARS-CoV-2 NCGM-05-2N strain (SARS-CoV-2^05-2N^) was isolated from nasopharyngeal swabs of a patient with COVID-19, who was admitted to the NCGM and was enrolled in this study (Case 1: Supplementary Table S1). Plasma or serum samples were collected from patients, and IgG fractions were purified using a spin column-based antibody purification kit (Cosmo Bio, Tokyo, Japan) according to the manufacturer’s instructions. In brief, serum or plasma was collected, heat-inactivated for 30 min at 56 °C, and spin columns were centrifuged at 3500 rpm for 5 min. IgG fractions in supernatants were eluted and collected.

### Antiviral assays

VeroE6^TMPRSS2^ cells were seeded in 96-well microtiter culture plates (1 × 10^4^ cells/well). On the following day, the virus (SARS-CoV-2^05-2N^) was mixed to each of the purified IgG fractions, incubated for 20 min at 37 °C, and the mixture containing virus was inoculated to the cells at a multiplicity of infection (MOI) of 0.01. After culturing the cells for 3 days, the levels of cytopathic effect (CPE) observed in SARS-CoV-2-exposed cells were determined using the WST-8 assay employing Cell Counting Kit-8 (Dojindo, Kumamoto, Japan). Cell culture supernatants were also harvested, viral RNA was extracted using QIAamp Viral RNA Mini Kit (QIAGEN, Hilden, Germany), and quantitative RT-PCR (RT-qPCR) was performed using One Step PrimeScript III RT-qPCR Mix (Takara Bio, Shiga, Japan) following the manufactures’ instructions. The primers and probe used for detecting SARS-CoV-2 envelope^16^ were: 5′-ACT TCT TTT TCT TGC TTT CGT GGT-3′ (forward), 5′-GCA GCA GTA CGC ACA CAA TC-3′ (reverse) and 5′-FAM-CTA GTT ACA CTA GCC ATC CTT ACT GC-BHQ1-3′ (probe).

### Immunocytochemistry

VeroE6^TMPRSS2^ or VeroE6 cells seeded in 96-well microtiter culture plates and cultured with or without SARS-CoV-2^05-2N^ exposure and in the presence or absence of IgG fractions were fixed with 4% paraformaldehyde in PBS for 15 min, washed with PBS (300 µL/well) three times, and blocked with a blocking buffer (10% goat serum, 1% BSA, 0.3% Triton X-100, and PBS 1X) for 1 h. After removing the blocking buffer, the cells were immediately stained (overnight at 4 °C) with a primary antibody: a convalescent IgG fraction from a patient (Case 8) isolated as described above. The stained cells were washed with PBS (300 µL/well) and the cells were incubated with the secondary antibody: goat polyclonal anti-human-IgG-Alexa Fluor 488 Fab fragment antibody (Jackson ImmunoResearch Laboratories, Inc, West Grove, PA, USA), together with Texas Red™-X dye conjugated Phalloidin (Invitrogen/Thermo Fisher Scientific) for filamentous-actin visualization for 2 h. After washing the cells with PBS (300 µL/well) three times, DAPI solution (Invitrogen/ Thermo Fisher Scientific) in PBS (50 µL/well) was added to stain nuclei. Signals were acquired with Cytation 5 Cell Imaging Multi-Mode Reader (BioTek, Winooski, VT, USA).

### Enzyme-linked immune sorbent assay (ELISA) for SARS-CoV-2-binding human IgG

The amounts of SARS-CoV-2-spike-protein-S1-domain (SARS-CoV-2-S1)-binding human IgG in the patient’s plasma or serum were measured using commercial ELISA kits [Anti-SARS-CoV-2 ELISA (IgG) (EUROIMMUN AG, Luebeck, Germany)] according to the manufacturer's instructions. The reagent wells of the ELISA were coated with the S1 domain of the spike protein of SARS-CoV-2 expressed recombinantly in the human cell line HEK 293. The results were evaluated and are shown by calculating ratios between the extinction of the sample and calibrator, and 1.1 was set as a cut-off value for positive results and all values < 1.1 were considered negative.

## Supplementary Information

Supplementary Information
