# Supplementary material for: Neutralization of SARS-CoV-2 with IgG from COVID-19-convalescent plasma
Source: Sci Rep. 2021 Mar 10;11:5563. doi: 10.1038/s41598-021-84733-5 (PMC7946899; doi:10.1038/s41598-021-84733-5)
Supplement: Supplementary file 1 — Supplementary Information [file 41598_2021_84733_MOESM1_ESM.pdf]

Supplemental Information

Neutralization of SARS-CoV-2 with IgG from COVID-19-convalescent plasma

Kenji Maeda[1*], Nobuyo Higashi-Kuwata[1], Noriko Kinoshita[2], Satoshi Kutsuna[2], Kiyoto Tsuchiya[3], Shin-ichiro Hattori[1], Kouki Matsuda[1], Yuki Takamatsu[1], Hiroyuki Gatanaga[3], Shinichi Oka[3], Haruhito Sugiyama[4], Norio Ohmagari[2], and Hiroaki Mitsuya[1,5,6*]

[1]Department of Refractory Viral Infections, National Center for Global Health and Medicine (NCGM) Research Institute, Tokyo, Japan. [2]Disease Control and Prevention Center (DCC), NCGM, Tokyo, Japan. [3]AIDS Clinical Center, NCGM, Tokyo, Japan. [4]Respiratory Medicine, NCGM Center Hospital, Tokyo, Japan. [5]Experimental Retrovirology Section, HIV and AIDS Malignancy Branch, National Cancer Institute, National Institutes of Health, Bethesda, Maryland, USA. [6]Department of Clinical Sciences, Kumamoto University Hospital, Kumamoto, Japan.

Supplementary Table S1. Detailed characteristics for 11 patients (Cases 1~11)

Patient No.	Age	Sex	Nationality	Chronic/Underlying diseases	Disease severity*1	Respiratory support	Outcome (days of discharge after the disease onset)	Day of PCR-negative confirmed	Days of sample collection after the disease onset
Case 1	70	M	Australian	Hypertension<br>Hyperlipidemia<br>Prostate cancer (after recovery)	Mild*2	None	Discharge (25)	13	1, 9
Case 2	61	M	Japanese	Hyperlipidemia<br>Arrhythmia (WPW)	Moderate	None	Discharge (33)	33	7, 23
Case 3	29	F	Japanese	-	Moderate	None	Discharge (39)	29	29, 66
Case 4	53	M	Japanese	-	Severe	Oxygen inhalation	Discharge (18)	16	11, 21, 35
Case 5	33	F	Chinese	-	Severe	Oxygen inhalation	Discharge (27)	24	8, 22, 28
Case 6	50	F	Japanese (Naturalized)	-	Severe	Oxygen inhalation	Discharge (15)	15	10, 29, 60
Case 7	61	M	Japanese	Hypertension<br>Diabetes	Severe	Oxygen inhalation	Discharge (21)	20	14, 33, 66
Case 8	83	M	Chinese Canadian	Hypothyroidism<br>Hypertension<br>Gout	Severe	Oxygen inhalation	Discharge (43)	36	18, 47
Case 9	40	M	Japanese	Diabetes<br>Syphilis (after treatment)	Critical	Ventilator	Discharge (29)	29	10, 23, 45
Case 10	74	M	Japanese	Diabetes<br>Hypertension	Critical	Ventilator + ECMO*3	Deceased (45)	Deceased	8, 16, 36
Case 11	63	M	American	Obesity	Critical	Ventilator + ECMO*3	Discharge (93)	56	8, 16, 36, 48, 65

*1For disease severity definitions, see footnote to Table 1. *2For Case 1, the presence of SARS-CoV-2 was confirmed with RNA-qPCR tests with nasal swabs (172,284 and 3,759 SARS-CoV-2 RNA copies/µl on days 1 and 3 following the disease onset, respectively).

Figure Legends

Supplementary Figure S1. Immunocytochemistry of SARS-CoV-2-exposed VeroE6 cells cultured in the presence of IgG fractions from Case 6. VeroE6 cells were exposed to SARS-CoV-$2^{05\text{-}2N}$ and cultured in the presence of various amounts of IgG fraction from Case 6, incubated for 5 days, and examined with immunocytochemistry. Merged images, which are composed of images obtained from 3-color fluorescence are shown: viral antigens, F-actin, and nuclei stained in green, red, and blue, respectively. The upper left inset shows the image of VeroE6 cells cultured alone, while the upper right the image of VeroE6 cells exposed to SARS-CoV-$2^{05\text{-}2N}$ and cultured in the absence of IgG. Lower insets show the images of VeroE6 cells exposed to SARS-CoV-$2^{05\text{-}2N}$ and cultured in the presence of COVID-19-convalescent IgG from Case 6 (29 days after the disease onset) at 0.8, 4, and 20 μg/ml.

Supplementary Figure S2. Definition of the Neutralizing Unit. (A) IgG fractions from a patient D008 potently blocked the infectivity of the virus, while IgG fractions collected from other patients (D005, D006 and D009) only partially blocked the infectivity as determined using the cytopathicity. **(B)** The images of $\text{VeroE6}^{\text{TMPRSS2}}$ cells under immunocytochemistry and light microscopy. The left upper and lower panels show the cells cultured alone and the right upper and lower panels show the cells exposed to SARS-CoV-$2^{05\text{-}2N}$ and cultured in the absence of IgG, whose images were captured under immunocytochemistry and light microscopy, respectively. **(C)** One neutralizing unit was defined as the minimum IgG concentration, which completely block the infectivity and replication of SARS-CoV-$2^{05\text{-}2N}$ in $\text{VeroE6}^{\text{TMPRSS2}}$ cells. The IgG fraction from patient D008 completely blocked the infectivity and replication at 100 μg/ml and was determined to have one neutralizing unit/ml (1 NU/ml) at 100 μg/ml.

Supplementary Figure S3. COVID-19-convalescent IgG fractions inhibit SARS-CoV-2 infection. (A) Inhibition of the cytopathic effect of SARS-CoV-$22^{05\text{-}2N}$ by convalescent IgG fractions from eleven patients. SARS-CoV-$2^{05\text{-}2N}$ mixed with an IgG fraction from each of the eleven COVID-19-convalescent patients upon admission (blue) and upon or after discharge (red) was inoculated to $\text{VeroE6}^{\text{TMPRSS2}}$ cells, cultured for 3 days, and the changes in the survivability of the cells were determined using the WST-8 assay. **(B)** Changes in viral copy numbers in the supernatants of $\text{VeroE6}^{\text{TMPRSS2}}$ cells exposed to the mixture of SARS-CoV-$2^{05\text{-}2N}$ and various concentrations of IgG fraction and cultured for 3 days are shown for all eleven cases. The days of sample collection (upon admission and a convalescent time point) following the disease onset [Case 1: day 1 and 9, Case 2: day 7 and 23, Case 3: day 29 and 66, Case 4: day 11 and 21, Case 5: day 8 and 22, Case 6: day 10 and 29, Case 7: day 14 and 33, Case 8: day 18 and 47, Case 9: day 10 and 23, Case 10: day 8 and 36, Case 11:day8 and 36].

Supplementary Figure S4. Immunocytochemistry of $\text{VeroE6}^{\text{TMPRSS2}}$ cells exposed to the mixture of SARS-CoV-$2^{05\text{-}2N}$ and IgG fraction from each COVID-19. (A) The neutralizing activity of IgG fractions (20 μg/ml) from plasma/serum samples of 9 cases is shown. Note that IgG fractions (20 μg/ml) from Cases 6 and 8

collected upon discharge virtually completely blocked the infection and cytopathicity of SARS-CoV-$2^{05\text{-}2N}$, but other IgG fractions showed moderate or partial neutralization activity. IgG fractions from Cases 1, 2, and 3 had least or no detectable neutralizing activity. **(B)** IgG fractions from Cases 7 and 8 collected upon admission moderately or partially blocked the infectivity of the virus, while their IgG fractions collected at later convalescent time point (33 and 47 days from the onset, respectively) potently and moderately blocked the infectivity. Note that 5-fold dilution of 4 μg/ml IgG of both Cases 8 and 11 down to 0.8 μg/ml IgG resulted in almost total loss of neutralizing activity. The days of sample collection (upon admission and a convalescent time point) following the disease onset [Case 1: day 1 and 9, Case 2: day 7 and 23, Case 3: day 29 and 66, Case 4: day 11 and 21, Case 5: day 8 and 22, Case 6: day 10 and 29, Case 7: day 14 and 33, Case 8: day 18 and 47, Case 9: day 10 and 23, Case 10: day 8 and 36, Case 11:day8 and 36].

Supplementary Figure S5. Examination of possible antibody-dependent enhanced (ADE) SARS-CoV-2 infection. (A) The numbers of viral RNA copies in the supernatants of $VeroE6^{TMPRSS2}$ cells exposed to the mixture of SARS-CoV-$2^{05\text{-}2N}$ and various concentrations of IgG fraction from Case 11 upon admission (that has no detectable neutralization activity) and that from Case 8 upon admission (that has moderate neutralization activity) were determined using RNA-PCR. Note that no significant increases in the RNA copy numbers were not seen, while the IgG fraction from Case 8 reduced the numbers at 20 μg/ml. **(B)** Immunocytochemistry of $VeroE6^{TMPRSS2}$ cells exposed to the mixture of SARS-CoV-$2^{05\text{-}2N}$ (at MOI of 0.005) and various concentrations of IgG fraction from Case 11. Note that the IgG fraction from Case 11 upon admission had no significant neutralization activity at concentrations used; however, his IgG fraction completely and moderately blocked the infectivity of the virus at 20 and 2 μg/ml, respectively. These immunocytochemistry data did not reveal the possible antibody-dependent enhanced infection of SARS-CoV-2.

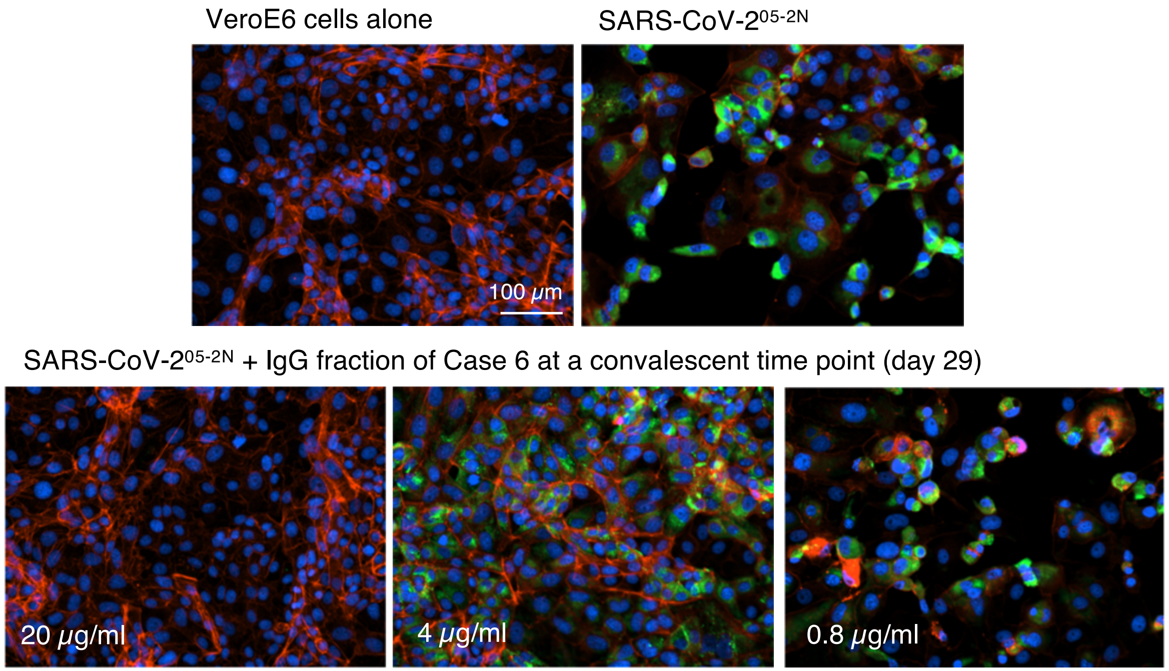

Supplementary Figure S1.

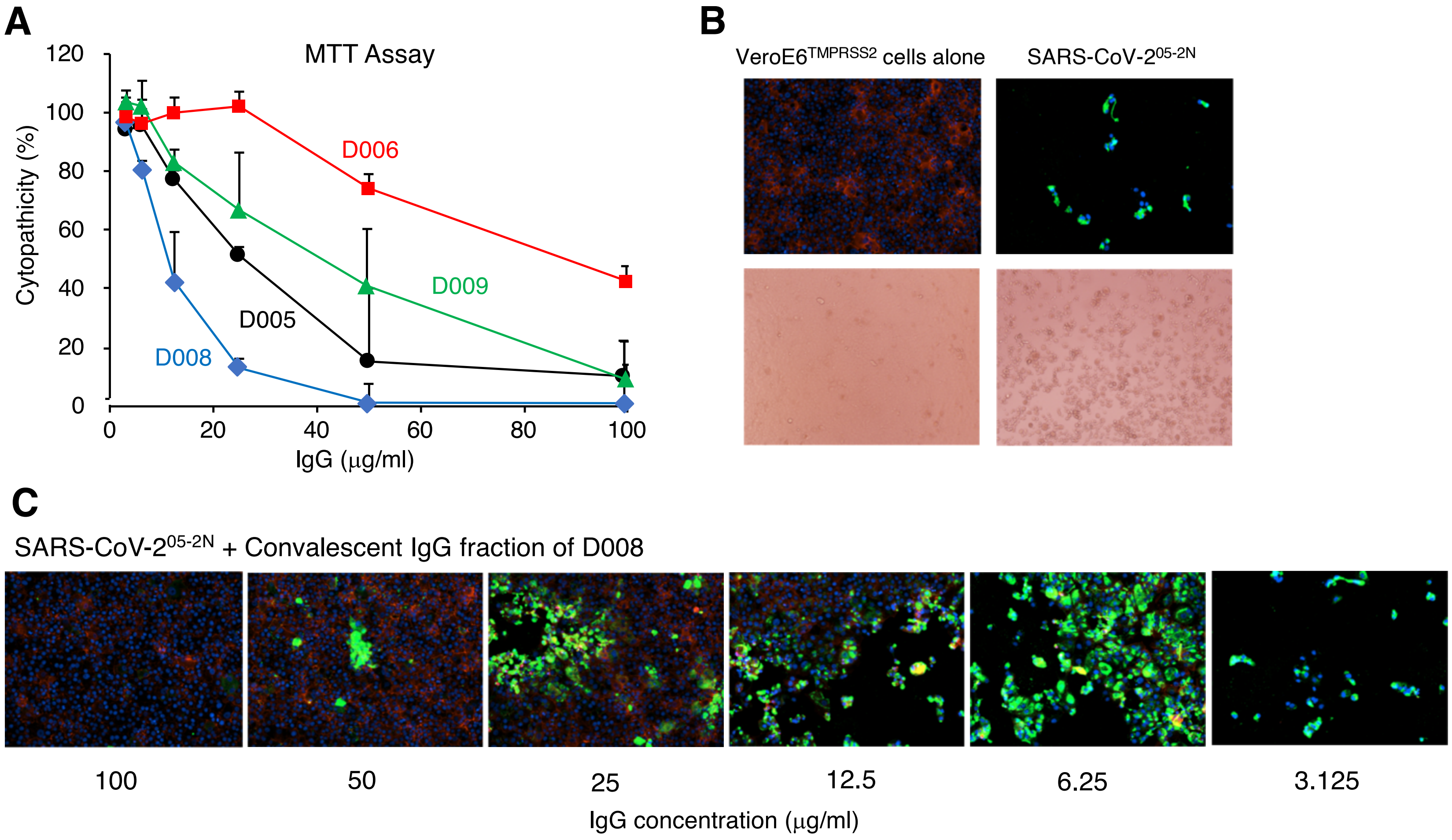

Supplementary Figure S2.

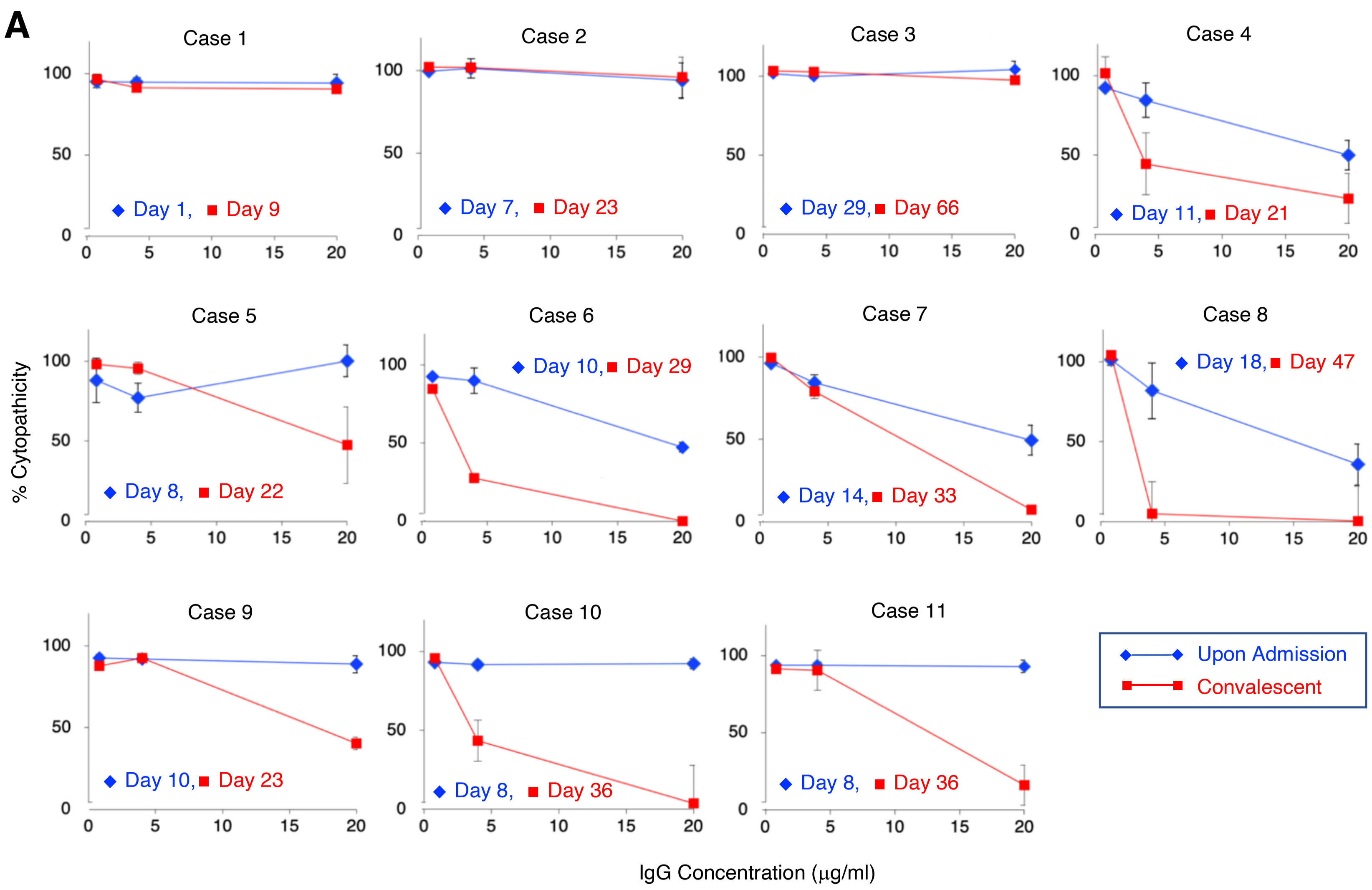

Supplementary Figure S3.

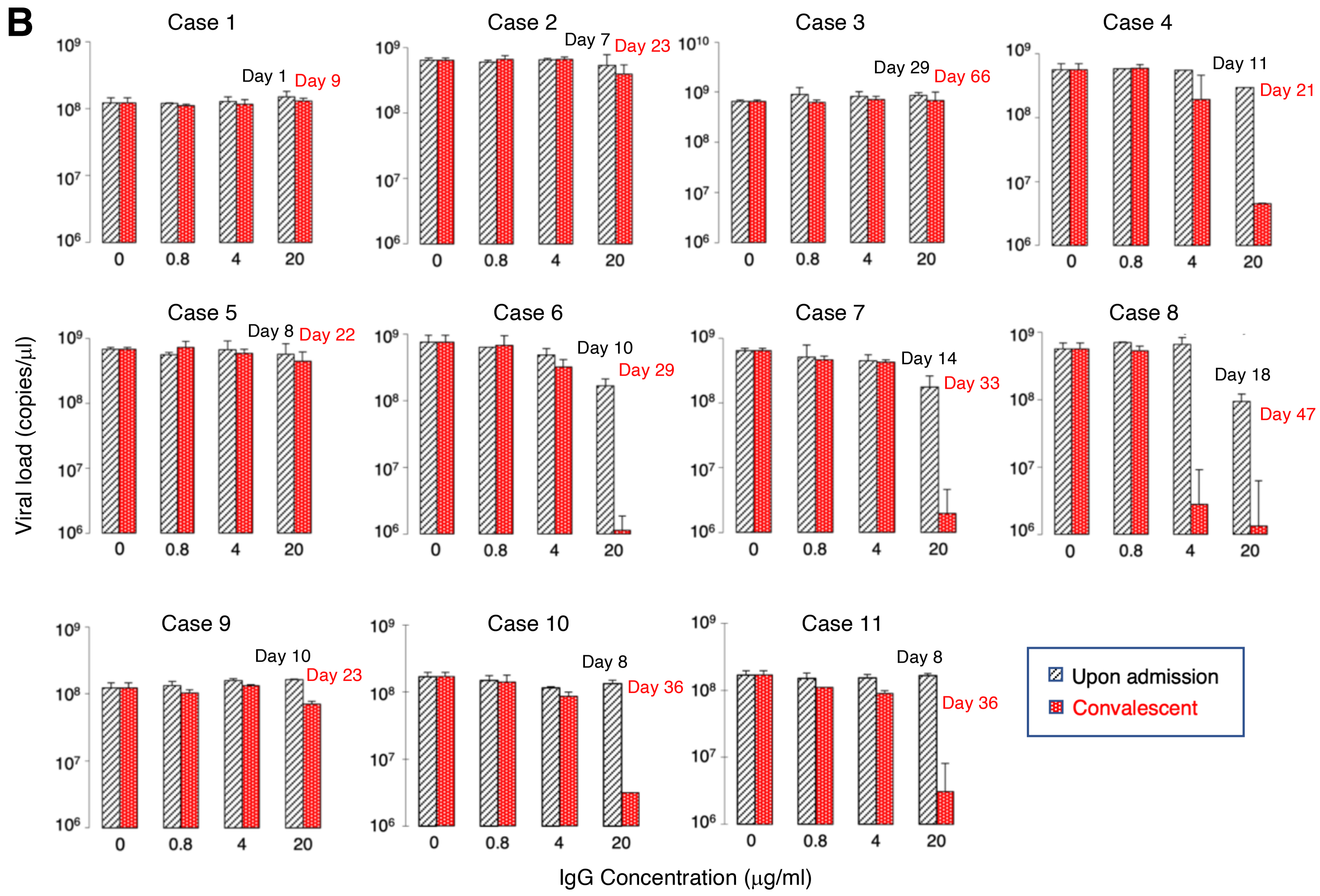

Supplementary Figure S3.

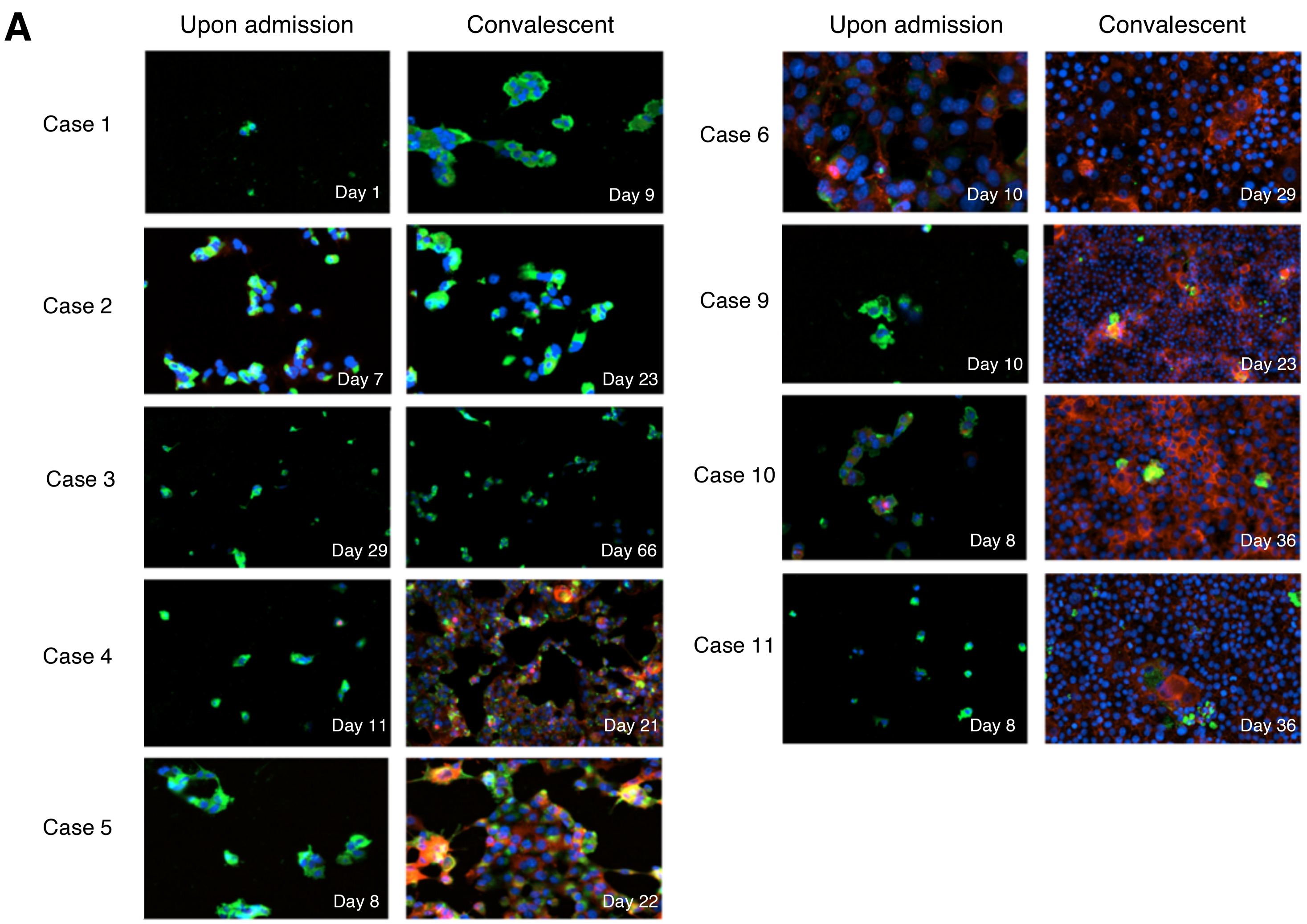

Supplementary Figure S4.

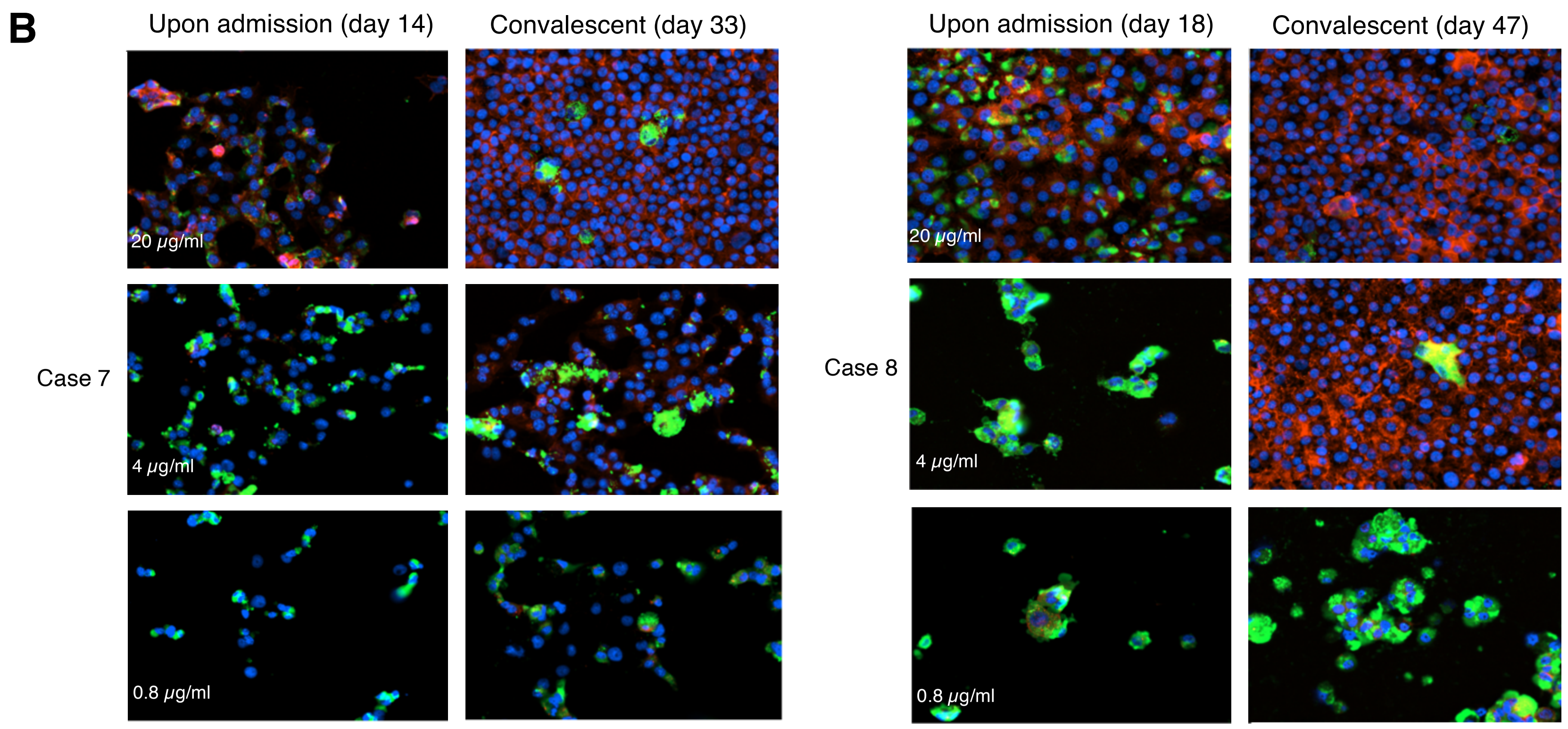

Supplementary Figure S4.

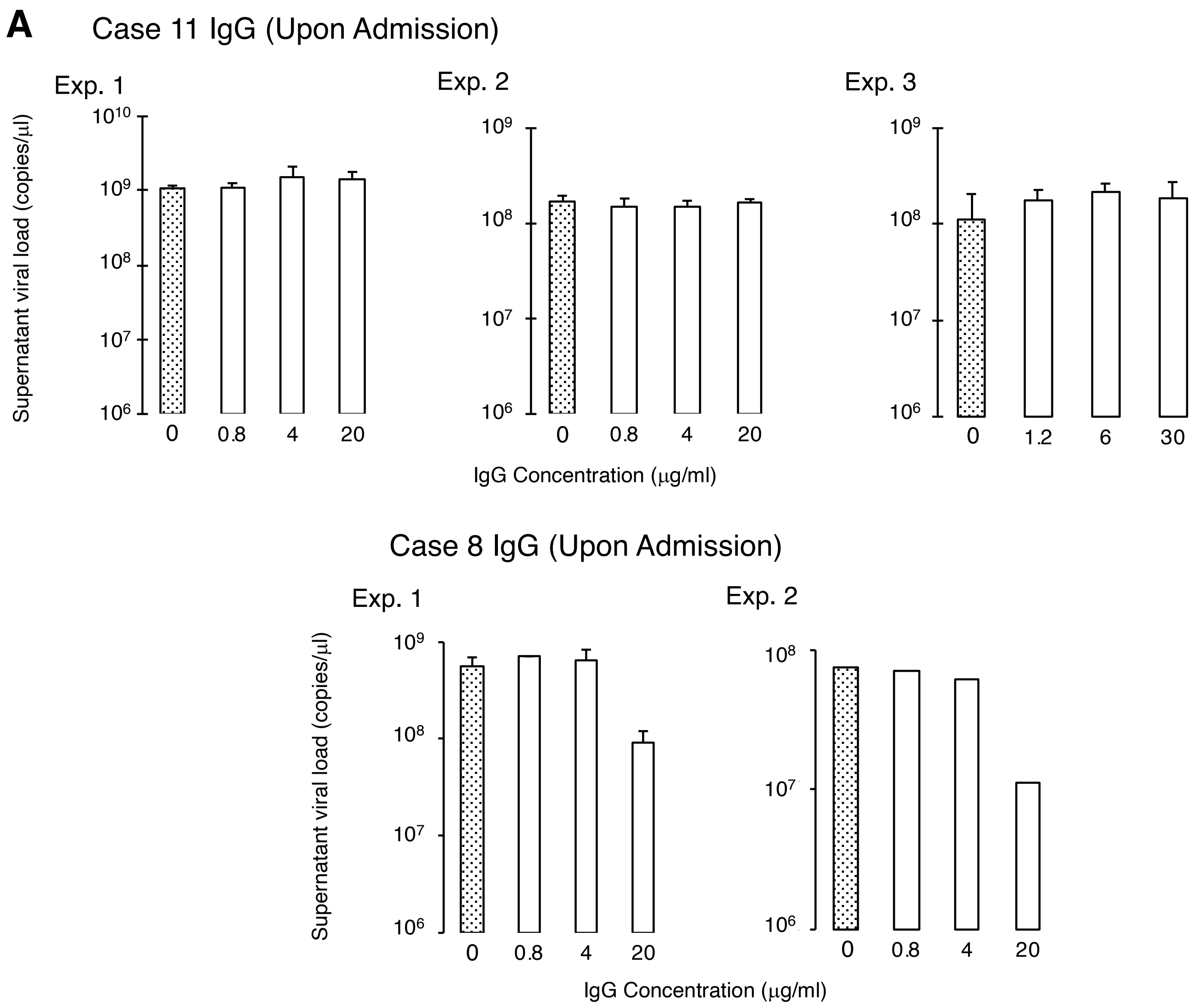

Supplementary Figure S5.
